# Associations of medicine use and ejection fraction with the coexistence of frailty and sarcopenia in a sample of heart failure outpatients: a cross-sectional study

**DOI:** 10.1186/s12872-023-03632-x

**Published:** 2023-12-05

**Authors:** Rui Valdiviesso, Teresa F. Amaral, Emília Moreira, Ana Rita Sousa-Santos, Mário Fernandes, Maria J. V. Aguiar, Sónia Martins, Luís F. Azevedo, Lia Fernandes, José Silva-Cardoso, Nuno Borges

**Affiliations:** 1 FCNAUP, Faculty of Nutrition and Food Sciences of the University of Porto, Rua Do Campo Alegre, 823, 4150-180 Porto, Portugal; 2 CINTESIS@RISE, MEDCIDS, Faculty of Medicine of the University of Porto, Porto, Portugal; 3 LAETA-INEGI / FEUP, Associated Laboratory of Energy, Transports and Aerospace, Institute of Science and Innovation in Mechanical and Industrial Engineering, Faculty of Engineering of the University of Porto, Porto, Portugal; 4 CINTESIS@RISE, Knowledge Management Unit, Faculty of Medicine of the University of Porto, Porto, Portugal; 5 TOXRUN, Toxicology Research Unit, University Institute of Health Sciences, CESPU, CRL, Gandra, Portugal; 6 CESPU, University Cooperative, CRL, Gandra, Portugal; 7 CINTESIS@RISE, Department of Clinical Neurosciences and Mental Health, Faculty of Medicine of the University of Porto, Porto, Portugal; 8 Psychiatry Service, University Hospital Center of São João, Porto, Portugal; 9 CINTESIS@RISE, Department of Medicine, Faculty of Medicine of the University of Porto, Porto, Portugal; 10 Department of Cardiology, University Hospital Center of São João, Porto, Portugal

**Keywords:** Heart Failure, Frailty, Sarcopenia, Coexistence, Left-ventricular ejection fraction, HFpEF, Pharmacotherapy

## Abstract

**Background:**

Frailty and sarcopenia have been extensively studied in heart failure (HF) patients, but their coexistence is unknown. The aim of this work is to describe the coexistence of these conditions in a sample of HF outpatients and its association with the use of medication and left-ventricular ejection fraction.

**Methods:**

Participants in this cross-sectional study were recruited from a HF outpatients’ clinic in northern Portugal. Frailty phenotype was assessed according to Fried et al. Sarcopenia was evaluated according to the revised consensus of the European Working Group on Sarcopenia in Older People.

**Results:**

A total of 136 HF outpatients (33.8% women, median age 59 years) integrated this study. Frailty and sarcopenia accounted for 15.4% and 18.4% of the sample, respectively. Coexistence of frailty and sarcopenia was found in 8.1% of the participants, while 17.6% had only one of the conditions. In multivariable analysis (*n* = 132), increasing age (OR = 1.13;95%CI = 1.06,1.20), being a woman (OR = 65.65;95%CI = 13.50, 319.15), having heart failure with preserved ejection fraction (HFpEF) (OR = 5.61; 95%CI = 1.22, 25.76), and using antidepressants (OR = 11.05; 95%CI = 2.50, 48.82), anticoagulants (OR = 6.11; 95%CI = 1.69, 22.07), furosemide (OR = 3.95; 95%CI = 1.07, 14.55), and acetylsalicylic acid (OR = 5.01; 95%CI = 1.10, 22.90) were associated with increased likelihood of having coexistence of frailty and sarcopenia, while using statins showed the inverse effect (OR = 0.06; 95%CI = 0.01, 0.30).

**Conclusions:**

The relatively low frequency of coexistence of frailty and sarcopenia signifies that each of these two conditions still deserve individual attention from health professionals in their clinical practice and should be screened separately. Being a woman, older age, having HFpEF, using anticoagulants, antidepressants, loop diuretics and acetylsalicylic acid, and not using statins, were associated with having concomitant frailty and sarcopenia. These patients can potentially benefit from interventions that impact their quality of life such as nutritional and mental health interventions and exercise training.

## Introduction

The syndrome of heart failure (HF) is a global public health problem in rapid expansion in both developed and developing countries [1]. As a condition associated with systemic multisystem dysfunction, HF is often accompanied by various comorbidities, which contribute to worst outcomes [2] and a heavier burden on health systems [3]. Two frequent comorbidities associated with HF are frailty and sarcopenia, with an overall estimated prevalence of 44.5% and 34.0% in elderly HF patients, respectively [4, 5]. Both conditions are associated with increased mortality and/or hospitalisation in HF patients [5–7].

Physical frailty is a state of vulnerability caused by the decline of reserve and function across multiple systems, which compromises the ability of coping with external stressors. As described by Fried et al., the frailty phenotype is present when three or more of the following criteria are met: low muscle strength; low physical performance; low physical activity; exhaustion and involuntary weight loss [8]. Sarcopenia is a systemic muscle disease, characterized by low muscle strength and quantity or quality. Low physical performance adds to the severity of the disease [9].

Frailty and sarcopenia are two distinct entities. However, in many cases, frail individuals are also sarcopenic, as low muscle strength and physical performance are common definitions. Notwithstanding to this, frailty remains a much wider concept that can encompass sarcopenia to a partial degree, but also components of mental state, changes in body weight and usual physical activity [8, 9]. In HF, frailty and sarcopenia also have different epidemiological behaviours: while frailty seems to be more prevalent in older patients with heart failure with preserved ejection fraction [10], sarcopenia affects HF patients irrespective of their phenotype [7, 11].

It has been postulated that HF, frailty, and sarcopenia share many common pathophysiologic characteristics, which include metabolic imbalance, systemic inflammation, mitochondrial dysfunction, oxidative stress, and raised levels of interleukine-6. These endocrine and metabolic abnormalities result not only in cardiac alterations but also in the loss of muscle mass and in the impairment of physical function, thus generating a vicious circle of disability [12, 13]. Despite the described relations between HF, frailty and sarcopenia, their coexistence was never, to our knowledge, reported in the literature.

Treating frailty and sarcopenia remains a challenge. Evidence regarding pharmacological therapies aimed exclusively at frailty is inconclusive or related to single-drug interventions on particular aspects of the syndrome [14]. It is known that polypharmacy is common in frail patients and is associated with worst outcomes, including the incidence of pre-frailty [15]. Therefore, one of the many challenges in managing frailty in HF is associated with the fact that polymedication is almost ubiquitous in HF patients, as guideline recommendations towards pharmacotherapy include a combination of medicines aimed at cardiovascular treatment and at the many HF comorbidities [2]. Similarly, evidence regarding drug treatments for sarcopenia in HF is still inconclusive and warranting further study [16]. On the other hand, some medicines commonly used in HF patients are known to be associated with changes in muscle health, either favourable or deleterious, thus impacting sarcopenia and/or frailty, but the evidence regarding the effect on muscle of medicines such as aldosterone antagonists, angiotensin receptor blockers, metformin, statins, and sodium-glucose co-transporter 2 inhibitors remains controversial [17].

The goal of the present study is, therefore, to describe the coexistence of frailty and sarcopenia in HF patients. We believe that studying this concomitance and associated factors should allow for identifying which individuals are at increased risk of accumulating health outcomes. Hence, as left-ventricular ejection fraction (LVEF) is an important defining criterion of HF and its outcomes in relation to frailty and sarcopenia, and pharmacologic therapies may impact these two conditions, we also aim to describe the association between these clinical variables and the co-occurrence of frailty and sarcopenia.

## Methods

The data that supports this cross-sectional study were collected between September 2017 and July 2018 in a HF outpatients’ clinic of a northern Portuguese university hospital, regarding a population of 537 potentially eligible participants, estimated from a study developed in a similar period on the same setting [18]. Participants were randomly selected from the daily physicians’ appointments lists.

**Fig. 1:**
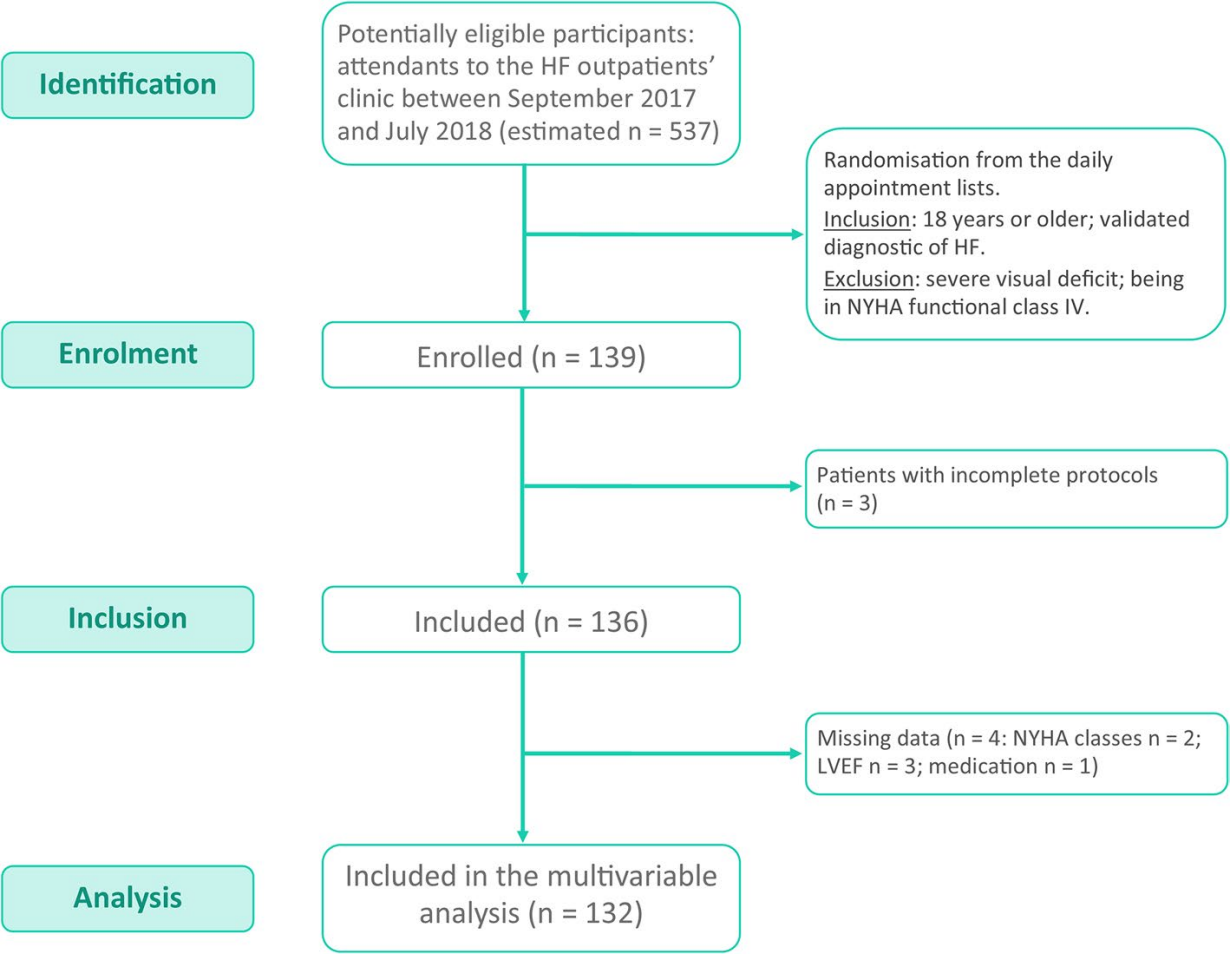
Flow diagram of the study

Inclusion and exclusion criteria were applied at the recruitment stage: participants were included if they were 18 years or older and had a clinically-validated diagnosis of HF according to the European Society of Cardiology (ESC) [2]; patients with severe visual impairment were excluded, as well as those within the NYHA (New York Heart Association) functional class IV, for their difficulty in complying with the research protocol. Figure 1 illustrates the flow diagram of the study.

Clinical data were collected during appointments with cardiologists. Medical records were also reviewed. The type of the disease was classified as heart failure with reduced ejection fraction (HFrEF), heart failure with mildly-reduced ejection fraction (HFmrEF) and heart failure with preserved ejection fraction (HFpEF), whenever patients presented LVEF < 40%, 40–50% or ≥ 50%, respectively [2]. Gathered data also included NYHA functional classes, incidental cardiac infarction, atrial fibrillation, diabetes, and medicines. Polypharmacy was defined as the concomitant daily use of five or more medicines [19].

Anthropometrical measurements were carried out by a registered nutritionist as described elsewhere [20], and include standing height, weight, triceps skinfold thickness, mid-upper arm girth, calf circumference and mid-upper arm muscle circumference.

Frailty phenotype was evaluated according to Fried et al. [8], as the occurrence of three or more of the following criteria: low strength, slow gait, exhaustion, low physical activity and unintentional weight loss. Methods for assessing each criterion for this study can be found in a previous work [20].

Sarcopenia was diagnosed according to the revised consensus of the European Working Group on Sarcopenia in Older People (EWGSOP2) [9]. Grip strength was measured according to the instructions of the American Society of Hand Therapists [21], using a Jamar Plus + digital hand dynamometer (Sammons Preston, USA). The average of three maximum compressions of the non-dominant hand was used. Low strength was defined as < 27 Kgf for men and < 16 Kgf for women [22]. Low muscle quantity was defined as mid-upper arm muscle circumference < 21.1 cm for men and < 19.2 cm for women [23], or as calf circumference < 31 cm [24].

### Statistics

The sample was described according to the presence of frailty and sarcopenia and to the co-occurrence of frailty and sarcopenia, categorised as: “*none of the conditions*”; “*one of the conditions*”; “*both conditions*”. Continuous variables were tested for normality using the Shapiro–Wilk test and were compared using parametric tests for variables with normal distribution and non-parametric tests for variables with skewed distribution. Values are respectively indicated in mean (M) and standard deviation (SD) and in median (Md) and interquartile range (IQR). Categorical variables were compared using the Qui-square or the Fisher exact tests, as adequate. Results are presented in number of individuals (n) and percentage (%).

An ordinal logistic regression was carried out to assess associations between the independent variables and the co-occurrence of frailty and sarcopenia as a dependent variable increasingly ordered regarding the number of conditions, from “*none of the conditions*” to “*one of the conditions*” to “*both conditions*”. The proportional odds model included the following predictors: continuous age; sex; asymptomatic patients within NYHA Class I vs. Classes II and III; patients with HFpEF vs. HFrEF and HFmrEF, and the use of medication (angiotensin-converting enzyme inhibitors, beta blockers, aldosterone antagonists, statins, furosemide, sacubitril + valsartan, ivabradine, thiazide diuretics, acetylsalicylic acid, nitrates, antidepressants, anxiolytics, digoxin, antiarrhythmic medicines, anticoagulants). A total of four participants were excluded from the multivariable analysis, due to missing values for NYHA functional classes (*n* = 2), LVEF (*n* = 3) and medication (*n* = 1). Crude and adjusted cumulative odds ratios (OR) and respective 95% confidence intervals (95% CI) were calculated. The test of parallel lines was used to evaluate the proportional odds assumption of the model. All tests were performed for a level of significance of *p* = 0.050. SPSS ver. 29 (IBM, USA) was used to execute all statistical analysis.

## Results

**Fig. 2:**
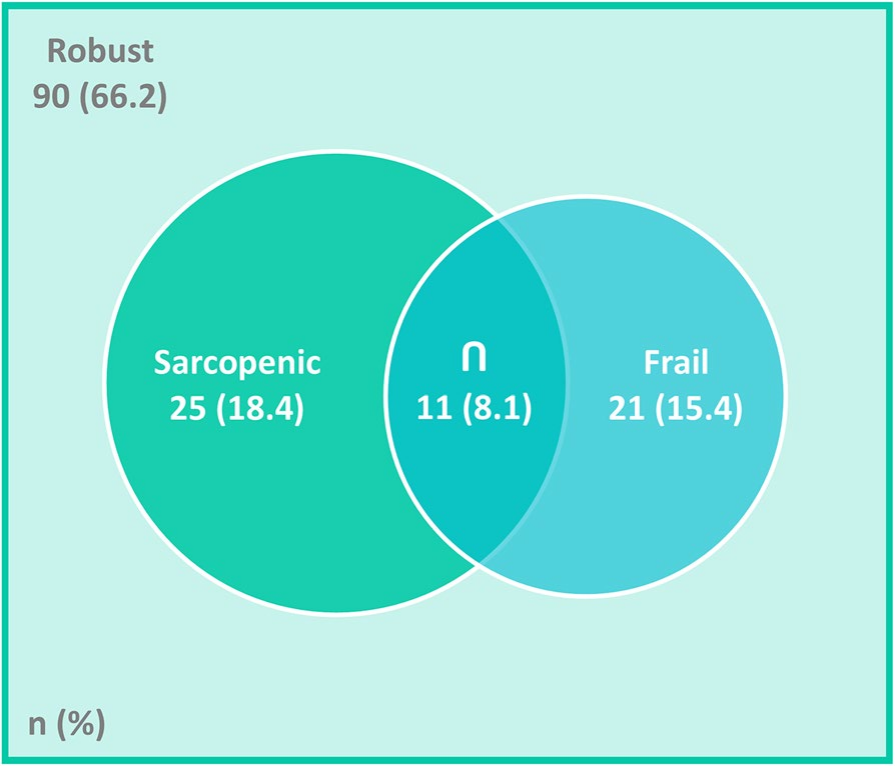
Venn diagram of the frequencies of frailty, sarcopenia, and the coexistence of both

A total of 136 HF outpatients (33.8% women, aged 24–81 years, median age 59 years) integrated this study. Figure 2 depicts a Venn diagram of the co-occurrence of frail and sarcopenic individuals in this sample, and of those who accumulated both conditions: 21 (15.4%) participants were frail and 25 (18.4%) were sarcopenic. The number of patients with concomitant frailty and sarcopenia was 11 (8.1%).

The characteristics of the sample regarding the presence of sarcopenia or frailty are presented in Table 1. Within sarcopenic participants, 44% were frail; 52% of frail participants were sarcopenic. Being a woman and having a higher usage of furosemide were the only common significant associations in frail and sarcopenic individuals. Frail patients were more likely to have less school years, to be at higher NYHA classes and to have higher frequency of prescription of antiarrhythmic medicines, anticoagulants, and antidepressants than non-frail ones. Sarcopenic patients were more likely to be older than non-sarcopenic ones, and to have lower usage of statins.

**Table 1:** Characterisation of the sample regarding frailty and sarcopenia

	**Conditions**					
	**Frailty**			**Sarcopenia**		
	**Normal + pre-frail** (*n* = 115)	**Frail** (*n* = 21)	***p*-values**	**Normal** (*n* = 111)	**Sarcopenic** (*n* = 25)	***p*-values**
Age, years, Md (IQR)	58.0 (49.0, 67.0)	64.0 (49.5, 71.5)	0.117	58.0 (49.0, 67.0)	67.0 (52.0, 70.5)	0.038
Age intervals, n (%)			0.146			0.009
< 65 years	79 (68.7)	11 (52.4)		79 (71.2)	11 (44.0)	
≥ 65 years	36 (31.3)	10 (47.6)		32 (28.8)	14 (56.0)	
Sex, n (%)			0.003			< 0.001
Women	33 (28.7)	13 (61.9)		24 (21.6)	22 (88.0)	
Men	82 (71.3)	8 (38.1)		87 (78.4)	3 (12.0)	
School years, Md (IQR)	9.0 (4.0, 12.0)	4.0 (4.0, 9.0)	0.023	9.0 (4.0, 12.0)	4.0 (4.0, 12.0)	0.399
NYAH classes, n (%)			0.003			0.189
Class I	45 (38.8)	2 (9.5)		42 (38.5)	5 (20.0)	
Class II	54 (47.8)	11 (52.4)		49 (45.0)	16 (64.0)	
Class III	14 (12.4)	8 (38.1)		18 (16.5)	4 (16.0)	
LVEF categories, n (%)			0.165			0.203
HFrEF	58 (51.3)	8 (40.0)		56 (51.9)	10 (40.0)	
HFmrEF	33 (29.2)	4 (20.0)		31 (28.7)	6 (24.0)	
HFpEF	22 (19.5)	8 (40.0)		21 (19.4)	9 (36.0)	
Polypharmacy, n (%)			0.593			0.127
< 5 medicines/day	30 (26.1)	4 (19.0)		31 (27.9)	3 (12.0)	
≥ 5 medicines/day	85 (73.9)	17 (81.0)		80 (72.1)	22 (88.0)	
Medicines, n (%)						
ACE inhibitors	90 (78.3)	16 (76.2)	0.781	87 (79.1)	19 (76.0)	0.734
Beta blockers	109 (94.8)	20 (95.2)	0.931	106 (96.4)	23 (92.0)	0.339
Aldosterone antagonists	81 (70.4)	10 (47.6)	0.041	77 (70.0)	14 (56.0)	0.178
Statins	78 (67.8)	11 (52.4)	0.171	77 (69.4)	12 (48.0)	0.042
Furosemide	38 (33.0)	13 (61.9)	0.012	37 (33.6)	14 (56.0)	0.037
Sacubitril/valsartan	16 (13.9)	0 (0.0)	0.132	13 (11.7)	3 (12.0)	1.000
Ivabradine	20 (17.4)	2 (9.5)	0.526	18 (16.2)	4 (16.0)	1.000
Thiazide diuretics	7 (6.1)	1 (4.8)	1.000	7 (6.3)	1 (4.0)	1.000
Acetylsalicylic acid	29 (25.2)	6 (28.6)	0.746	30 (27.0)	5 (20.0)	0.615
Nitrates	13 (11.3)	2 (9.5)	1.000	11 (9.9)	4 (16.0)	0.477
Digoxin	9 (7.8)	3 (14.3)	0.397	11 (9.9)	1 (4.0)	0.695
Antiarrhythmic drugs	10 (8.7)	6 (28.6)	0.009	12 (10.8)	4 (16.0)	0.495
Anticoagulants	35 (30.4)	12 (57.1)	0.018	38 (34.2)	9 (36.0)	0.867
Antidepressants	18 (15.7)	9 (42.9)	0.004	22 (19.8)	5 (20.0)	0.984
Anxiolytics	31 (27.8)	5 (23.8)	0.764	30 (27.0)	6 (24.0)	0.757
Diabetes, n (%)	34 (29.8)	5 (23.8)	0.794	33 (30.0)	6 (24.0)	0.550
Myocardial infarction, n (%)	29 (25.7)	3 (15.0)	0.402	28 (25.7)	4 (16.7)	0.436
Atrial fibrillation, n (%)	13 (11.8)	6 (28.6)	0.083	16 (15.1)	3 (12.0)	1.000
BMI, Kg.m^−2^, M (SD)	29.2 (4.4)	29.5 (4.2)	0.930	29.5 (4.2)	28.0 (4.7)	0.532
BMI categories, n (%)			0.952			0.118
Underweight + normal	21 (18.3)	4 (19.0)		17 (15.3)	8 (32.0)	
Overweight	48 (41.7)	8 (38.1)		46 (41.4)	10 (40.0)	
Obese	46 (40.0)	9 (42.9)		48 (43.2)	7 (28.0)	
**Coexistence**						
Frailty	-	-	-	10 (9.0)	11 (44.0)	< 0.001
Sarcopenia	14 (12.2)	11 (52.4)	< 0.001	-	-	-

Results presented in number (n) and percentage (%), in mean (M) and standard deviation (SD), or in median (Md) and inter-quartile range (IQR)

*NYHA* New York Heart Association, *LVEF* Left-ventricular ejection fraction, *HFrEF* Heart failure with reduced ejection fraction, *HFmrEF* heart failure with mid-range ejection fraction, *HFpEF* heart failure with preserved ejection fraction, *ACE* angiotensin-converting enzyme, *BMI* body mass index. Missing values: LVEF = 3; NYHA = 2; medicines = 1; incidental stroke = 3; atrial fibrillation = 5

A description of the sample stratified by the number of concomitant conditions can be found in Table 2. Roughly three quarters of the sample were not frail nor sarcopenic and 17.6% had only one condition. Being a woman, being older than 65 years, having less schooling, being at a higher NYHA class, not being prescribed aldosterone antagonists and statins, and using furosemide were all factors related with being concomitantly frail and sarcopenic.

**Table 2:** Characterisation of the sample regarding the co-occurrence of frailty and sarcopenia

	**Number of conditions**, n (%)			***p*-value**
	**No conditions** 101 (74.3)	**One condition** 24 (17.6)	**Two conditions** 11 (8.1)	
Age, years, Md (IQR)	58.0 (48.5, 67.0)	59.5 (50.3, 67.8)	70.0 (50.0, 73.0)	0.065
Age categories, n (%)				0.021
< 65 years	71 (70.3)	16 (66.7)	3 (27.3)	
≥ 65 years	30 (29.7)	8 (33.3)	8 (72.7)	
Sex, n (%)				< 0.001
Women	21 (20.8)	15 (62.5)	10 (90.9)	
Men	80 (79.2)	9 (37.5)	1 (9.1)	
School years, Md (IQR)	9.0 (4.0, 12.0)	9.0 (4.0, 12.0)	4.0 (4.0, 4.0)	0.009
NYAH functional classes, n (%)				0.021
Class I	41 (41.4)	5 (20.8)	1 (9.1)	
Class II	46 (46.5)	11 (45.8)	8 (72.7)	
Class III	12 (12.1)	8 (33.3)	2 (18.2)	
LVEF categories, n (%)				0.194
HFrEF	51 (51.5)	12 (52.2)	3 (27.3)	
HFmrEF	29 (29.3)	6 (26.1)	2 (18.2)	
HFpEF	19 (19.2)	5 (21.7)	6 (54.5)	
Polypharmacy, n (%)				0.384
< 5 medicines/day	28 (27.2)	5 (20.8)	1 (9.1)	
≥ 5 medicines/day	73 (72.3)	19 (79.2)	10 (90.9)	
Medicines, n (%)				
ACE inhibitors	80 (80.0)	17 (70.8)	9 (81.8)	0.592
Beta blockers	97 (97.0)	21 (87.5)	11 (100)	0.143
Aldosterone antagonists	70 (70.0)	18 (75.0)	3 (27.3)	0.015
Statins	72 (72.0)	11 (45.8)	6 (54.5)	0.046
Furosemide	32 (32.0)	11 (45.8)	8 (72.7)	0.022
Sacubitril/valsartan	13 (13.0)	3 (12.5)	0 (0.0)	0.596
Ivabradine	17 (17.0)	4 (16.7)	1 (9.1)	0.927
Thiazide diuretics	6 (6.0)	2 (8.3)	0 (0.0)	0.827
Acetylsalicylic acid	27 (27.0)	5 (20.8)	3 (27.3)	0.844
Nitrates	11 (11.0)	2 (8.3)	2 (18.2)	0.794
Digoxin	9 (9.0)	2 (8.3)	1 (9.1)	0.999
Antiarrhythmic drugs	9 (9.0)	4 (16.7)	3 (27.3)	0.119
Anticoagulants	30 (30.0)	13 (54.2)	4 (36.4)	0.077
Antidepressants	16 (16.0)	8 (33.3)	3 (27.3)	0.124
Anxiolytics	28 (28.0)	5 (20.8)	3 (27.3)	0.799
Diabetes, n (%)	32 (32.0)	3 (12.5)	4 (36.4)	0.114
Myocardial infarction, n (%)	26 (26.0)	5 (22.7)	1 (9.1)	0.573
Atrial fibrillation, n (%)	12 (12.5)	5 (20.8)	2 (18.2)	0.529
BMI, Kg.m^−2^, M (SD)	29.6 (4.3)	27.4 (3.7)	30.1 (5.0)	0.068
BMI categories, n (%)				0.332
Underweight + normal	16 (15.8)	6 (25.0)	3 (27.3)	
Overweight	41 (40.6)	12 (50.0)	3 (27.3)	
Obese	44 (43.6)	6 (25.0)	5 (45.5)	

Results presented in number (n) and percentage (%), in mean (M) and standard deviation (SD), or in median (Md) and inter-quartile range (IQR)

*NYHA* New York Heart Association, *LVEF* Left-ventricular ejection fraction, *HFrEF* Heart failure with reduced ejection fraction, *HFmrEF* heart failure with mid-range ejection fraction, *HFpEF* heart failure with preserved ejection fraction, *ACE* angiotensin-converting enzyme, *BMI* body mass index. Missing values: LVEF = 3; NYHA = 2; medicines = 1; incidental stroke = 3; atrial fibrillation = 5

The multivariable analysis included 132 participants, 20 frail (15.2%) and 25 sarcopenic (18.9%), from whom 11 (8.3%) had both conditions. Results from the ordinal logistic regression are presented in Table 3. For every year increase in age, the cumulative odds of having more conditions increased by 13% (OR = 1.13; 95%CI = 1.06, 1.2). Women were much more likely to be allocated in higher categories of coexistence of frailty and sarcopenia than men (OR = 65.65; 95%CI = 13.50, 319.15). Patients with HFpEF were more likely to have an accumulation of conditions than those with reduced or mid-range LVEF (OR = 5.61; 95%CI = 1.22, 25.76). Regarding medication, the participants who used statins were less likely to be allocated in higher categories of co-occurrence of frailty and sarcopenia than those who were not statin users (OR = 0.06; 95%CI = 0.01, 0.30), while patients who were prescribed anticoagulants (OR = 11.05; 95%CI = 2.50, 48.82), antidepressants (OR = 11.05; 95%CI = 2.50, 48.82), furosemide (OR = 3.95; 95%CI = 1.07, 14.55), and acetylsalicylic acid (OR = 5.01; 95%CI = 1.10, 22.90) were more likely to accumulate conditions. No associations were found for the remaining 10 medicines nor for NYHA functional classification.

**Table 3:** Results from the ordinal logistic regression analysis regarding the cumulative number of conditions from none to one condition (frailty or sarcopenia), to two coexisting conditions (frailty and sarcopenia)

	**Unadjusted**		**Adjusted**	
	**OR (95% CI)**	***p*-value**	**OR (95% CI)**	***p*-value**
Age	1.03 (1.00, 1.07)	0.050	1.13 (1.06, 1.20)	< 0.001
Sex				
Men	1		1	
Women	10.81 (4.42, 26.49)	< 0.001	65.65 (13.50, 319.15)	< 0.001
LVEF				
HFrEF + HFmrEF	1		1	
HFpEF	2.09 (0.91, 4.80)	0.081	5.61 (1.22, 25.76)	0.026
NYHA Classification				
Classes II + III	1		1	
Class I	0.29 (0.11, 0.76)	0.012	1.32 (0.26, 6.79)	0.742
Medicines				
ACE inhibitors	0.82 (0.32, 2.07)	0.668	0.74 (0.09, 6.15)	0.783
Beta blockers	0.45 (0.09, 2.29)	0.340	0.52 (0.04, 6.84)	0.638
Aldosterone antagonists	0.51 (0.23, 1.32)	0.098	0.71 (0.17, 2.97)	0.879
Statins	0.42 (0.19, 0.93)	0.032	0.06 (0.01, 0.30)	0.001
Furosemide	2.76 (1.26, 6.08)	0.011	3.95 (1.07, 14.55)	0.039
Sacubitril/valsartan	0.64 (0.16, 2.46)	0.514	0.81 (0.05, 12.43)	0.879
Ivabradine	0.79 (0.27, 2.33)	0.669	0.82 (0.14, 4.79)	0.828
Thiazide diuretics	0.44 (0.05, 3.93)	0.464	0.04 (0.01, 1.52)	0.083
Acetylsalicylic acid	0.92 (0.37, 2.27)	0.856	5.01 (1.10, 22.90)	0.038
Nitrates	1.13 (0.35, 3.72)	0.837	0.20 (0.03, 1,38)	0.103
Digoxin	0.96 (0.25, 3.73)	0.954	0.21 (0.02, 1.85)	0.158
Antiarrhythmic drugs	2.33 (0.79, 6.86)	0.124	1.09 (0.16, 7.64)	0.928
Anticoagulants	1.88 (0.85, 4.14)	0.118	6.11 (1.69, 22.07)	0.006
Antidepressants	2.37 (0.95, 5.89)	0.063	11.05 (2.50, 48.82)	0.002
Anxiolytics	0.75 (0.30, 1.90)	0.545	0.26 (0.06, 1.17)	0.078

Results presented in cumulative odds ratio (OR) and 95% confidence intervals (95% CI)

*NYHA* New York Heart Association, *LVEF* Left-ventricular ejection fraction, *HFrEF* Heart failure with reduced ejection fraction, *HFmrEF* heart failure with mid-range ejection fraction, *HFpEF* heart failure with preserved ejection fraction, *ACE* angiotensin-converting enzyme. The reference category for medicines (OR = 1) is “not using the medicine”. Chi-square of final model fitness = 88.5, *p* < 0.001. Nagelkerke R-square = 63.5%. Test of parallel lines: *p* = 0.944

**Fig. 3:**
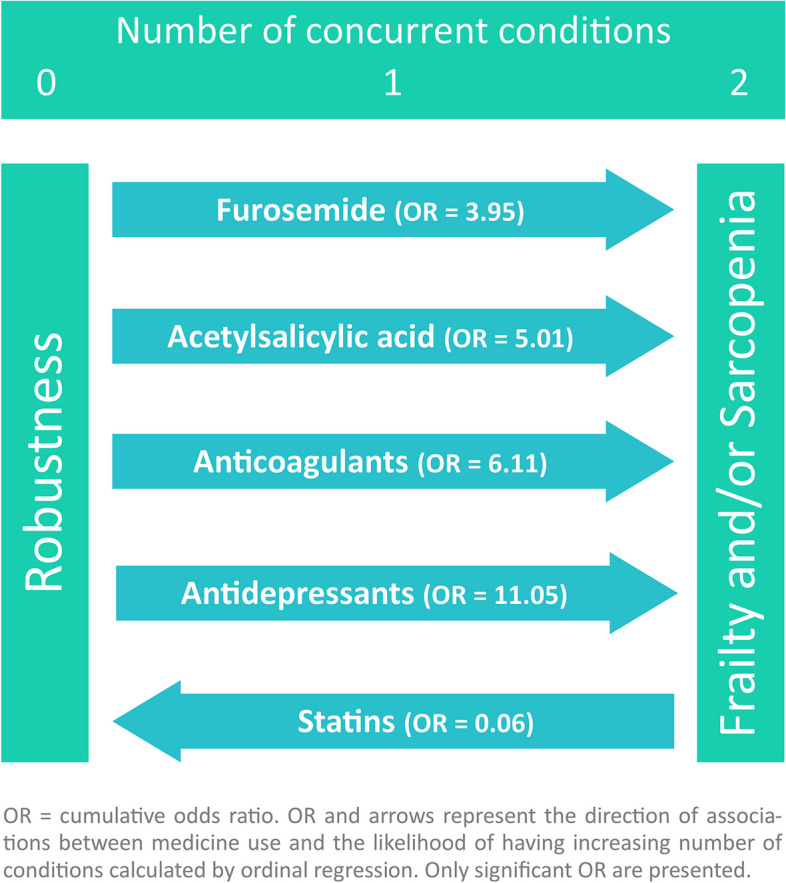
Direction of associations between medicine use and the coexistence of frailty and sarcopenia

The results regarding the associations between medicine use and coexistence of frailty and sarcopenia are summarised in Fig. 3.

## Discussion

### The coexistence of frailty and sarcopenia

Studies on the overlap of frailty and sarcopenia are unknown in HF patients and are scarce in other populations. Sousa-Santos et al. found a frequency of 2.2% of coexistence of these conditions in a sample of community-dwelling Portuguese older adults (*n* = 1454, age ≥ 65 years) [25]. Rasheedy & EL-Kawaly reported an overlap of 25.3% of frailty and sarcopenia in a sample of 206 hospitalized Egyptian patients with ages ≥ 60 years and with multiple comorbidities and diseases, including 21.4% of HF patients who were not stratified for the co-occurrence [26]. Both these studies used the same assessment methods (Fried et al. and EWGSOP2) for classifying frailty and sarcopenia as the present work. Ibrahim et al., using the Fried et al. and the previous EWGSOP criteria [27], found a frequency of 14% of overlapping between frailty and sarcopenia in a sample of older British patients hospitalised for acute disease (*n* = 233, age ≥ 70 years) [28]. The same defining methods were used by Gingrich et al. in a sample of 100 German inpatients older than 70 years, with a reported frequency of 19% of co-occurrence of frailty and sarcopenia [29]. The present study’s overlapping frequency of 8.1% seems to be placed between the reported percentage for community-dwelling older adults and data from hospitalised geriatric patients. The relatively low frequency of coexistence of frailty and sarcopenia signifies that each of these two conditions still deserve individual attention from health professionals in their clinical practice.

### The associations between medicine use and the coexistence of frailty and sarcopenia

The prescription of antidepressants is associated with the cumulative existence of frailty and sarcopenia in the present study. The association of depression with frailty is well established, with each condition being associated with increased prevalence and incidence of the other [30]. One of the five criteria for assessing FP according to Fried et al., is the presence of exhaustion [8], which is evaluated using two questions taken from the Center for Epidemiologic Studies of Depression scale [31] and in a previous work of this research group, we found that exhaustion was the most frequent criterion for defining frailty in this sample [20]. Sarcopenia also seems to be related with depressive symptoms [32–34]. The assessment of depressive symptoms and its association with frailty and sarcopenia in this sample is to be further studied, but we believe that these patients could benefit from mental health interventions to improve their quality of life.

In this study, the use of anticoagulants was a predictor of co-occurrence of frailty and sarcopenia. Frail patients with atrial fibrillation have higher risks of incidental stroke, mortality and duration of hospitalisation than non-frail ones [35] and anticoagulants can improve the prognosis of frail individuals. Despite this, the prescription of anticoagulants is usually restricted in frail patients due to the risk of falls and bleeding complications [36]. A meta-analysis by Oqab et al. comprising three studies on the prescription of anticoagulants in older adults with atrial fibrillation, concluded that frail patients were less likely to receive this medication when compared with non-frail ones [37]. In our bivariable analysis, both anticoagulant use and atrial fibrillation were not related with the categories of coexistence of frailty and sarcopenia, but frail patients were more likely to use anticoagulants. It is worth mentioning that the patients in this sample were not previously diagnosed for frailty nor sarcopenia, thus these conditions could not have been a factor for selective prescription of anticoagulants, while having atrial fibrillation was: 95% of the patients in this condition were prescribed anticoagulants (data not shown). This result deserves further study.

By contrast to anticoagulants and antidepressants, statins were associated with a reduced likelihood of accumulating frailty and sarcopenia. This finding enhances the results of a previous work by this research group, where statin use was associated with reduced odds of being sarcopenic, a relation that may be hypothetically attributable to the pleiotropic actions of this medication in neuromuscular health, possibly mediated by a better endothelial health [38]. Several biomarkers involved in oxidative stress and inflammation are likely to play a pathophysiologic role in sarcopenia and frailty [39]. Such is the case of microRNAs (miRNAs), which integrate cellular mechanisms affecting the functionality of mitochondria and muscle fibres [40]. Dysregulation of miRNAs is associated with the development of endothelial dysfunction [41], and statins have been shown the ability to modulate miRNAs expression [42]. Notably, miRNAs are also involved in the regulation of cardiomyocyte death, which is a central event in HF [43]. These observations can increment the understanding of the common pathophysiological origins of HF and sarcopenia.

Our results regarding the association of acetylsalicylic acid with higher odds of being frail and/or sarcopenic deserve further research, as cyclooxygenase inhibitors seem to have no effect on muscle mass and frailty status [44, 45].

As for the association of furosemide with the coexistence of frailty and sarcopenia, a paper by Nakano et al., reported an association between the use of loop diuretics and muscle wasting, assessed by arm and thigh circumferences, in patients with HF. The authors argue that this result may be attributable to the inhibitory effect of loop diuretics in the Na–K-Cl cotransporter NKCC1, thus downregulating skeletal muscle myogenesis [46]. Therefore, it is possible that the exposure to furosemide treatment has been causing muscle wasting in our sample. Discontinuation of loop diuretics in older HF patients has been recently discussed, and more research is warranted to account for the potential beneficial effects of withdrawing loop diuretics in frail HF patients [47].

The lack of associations regarding ACE-inhibitors and beta-blockers seems to be confirmed by the work of Abe et al., that reported an absence of interaction between renin–angiotensin–aldosterone system inhibitors and beta-blocker combination therapy and physical frailty in HFrEF and HFmrEF [48].

### The association of HFpEF with the coexistence of frailty and sarcopenia

In the present study, having HFpEF was associated with a very increased likelihood of having co-occurrence of frailty and sarcopenia. It is known that HFpEF patients are more likely to be frail compared with HFrEF patients [10], and sarcopenia may share a common pathophysiology with HFpEF and frailty [13, 49], which seems to confirm our results. Kinugasa and Yamamoto postulate that metabolic and endocrine abnormalities in sarcopenia, and to a greater extent, sarcopenic obesity, seem to be associated with the development of HFpEF. The authors suggest that the interplay between the pathophysiologic mechanisms of sarcopenia and obesity contribute to the onset of cardiovascular remodelling or diastolic dysfunction that leads to HFpEF [13]. Despite this, sarcopenia seems to contribute to mortality similarly in HFpEF and HFrEF [7].

HFrEF and HFpEF have different epidemiological and aetiological profiles: patients with preserved LVEF are typically older, more often women and have a higher propensity for having a history of atrial fibrillation and hypertension than patients with HFrEF, whereas deaths and hospitalisations for HFrEF are more likely to be related to cardiovascular events such as myocardial infarction [50, 51]. Consequentially, pharmacological treatment for HFrEF is mainly centred in the cardiac function, with demonstrated success for medicines such as ACE inhibitors, beta-blockers and sacubitril/valsartan [2]. Until recently, pharmacological treatments capable of reducing mortality and hospitalisation of HFpEF patients were inexistent, and treating these patients remained a challenge in cardiology [52], with therapeutical approaches usually limited to the management of symptoms and comorbidities and to the improvement of the quality of life [52, 53]. Novel evidence regarding the effect of sodium–glucose co-transporter 2 (SGLT2) inhibitors on reducing hospitalisation and cardiovascular death on HFpEF and HFmrEF patients has been emerging, and the ESC guidelines were recently updated to account for this fact [54].

The effect of SGLT2 inhibitors on reducing blood glucose levels, even in non-diabetic HF patients, may contribute to lower the risks and complications of frailty, as it has been shown that hyperglycaemia is associated with cognitive impairment [55] and with low physical function [56] in frail hypertensive older adults. Noteworthily, cognitive impairment and low physical performance are highly correlated in frail patients with acute myocardial infarction [57]. Mone et al. reported that the SGLT2 inhibitor empagliflozin improved cognitive and physical functions [58] and mRNAs signature of endothelial dysfunction in frail diabetic patients with HFpEF [59]. However, a recent meta-analysis revealed that the use of SGLT2 inhibitors may increase the risk of sarcopenia in patients with type-2 diabetes [60]. Therefore, patients using SGLT2 inhibitors should be carefully monitored for unwanted outcomes related with muscle health.

It is important to acknowledge that drug treatments for sarcopenic patients with HF are in the embryonic stage and still to be proven safe and effective [16] and no exclusive medicine therapy is known for addressing frailty in HF. Sarcopenia is also associated with reduced quality of life in patients with HFpEF [61]. For these reasons, interventions centred in nutrition and exercise training, alongside SGLT2 inhibitors, can potentially improve the quality of life of HFpEF patients with associated frailty and sarcopenia [13, 58, 62, 63].

### Limitations and strengths

Some limitations can be accredited to this exploratory study. First, causal associations cannot be inferred due to the cross-sectional nature of this work. Furthermore, the relatively small size of the sample might limit the interpretation of the results. It is also important to acknowledge that this study encompassed a sample of rather young HF outpatients. Apart from some rare exceptions concerning end-stage HF patients, other studies that focused on frailty and sarcopenia in HF populations were generally developed around much older samples, an aspect that must be contemplated when establishing comparisons between this work and others. Also, this study was developed in an outpatients’ setting, and it has been shown that the frequency of different HF phenotypes and patients’ genres can widely vary across setting [64]. The exclusion of the patients in NYHA functional class IV could be another source of bias, as it is possible that these individuals would be more likely to be classified as frail and/or sarcopenic. These limitations are likely to constraint the external validity of this study’s findings and a potential underestimation of frailty and/or sarcopenia in relation to other studies should be considered. Replicability of this study may also be limited due to recent changes in the guidelines that underlie the pharmacological treatments this sample was following at the time of the data collection, as recommendations towards the use of SGLT2 inhibitors in patients with HFpEF and HFmrEF were only yielded in 2023 [54]. Finally, muscle quantity was classified using anthropometric measurements, while EWGSOP2 recommends the use of other methods which were not available at the clinical setting this study was conducted in, such as dual energy x-ray absorptiometry or bioelectrical impedance. In these circumstances, the EWGSOP2 recommends the use of calf circumference [9], which is supported by a recent publication from Sousa-Santos et al., that showed a very high specificity (100%) of calf circumference to classify sarcopenia in relation to appendicular skeletal muscle mass measured using dual energy x-ray absorptiometry [65]. We additionally estimated muscle mass from mid-upper arm muscle circumference as this body part is usually free from oedema in HF patients, as discussed elsewhere [20].

We also recognise strengths in this study: sarcopenia was diagnosed according to the latest European consensus [9], and we assessed physical frailty using the Fried phenotype [8], as recommended by Denfeld et al. [4], as it is the most commonly used frailty definition in HF populations, thus allowing for comparisons between different studies. Moreover, this is, to our knowledge, the first work that describes the coexistence of sarcopenia and frailty in HF patients and associates the co-occurrence of these conditions to clinical variables such as LVEF and medicine use. However, the burden of the coexistence of frailty and sarcopenia remains unknown. It would be meaningful to investigate the outcomes resulting from the cumulative effects of both these conditions in HF patients, namely in mortality and hospitalisation. Additional studies should focus on the intervention on frail and/or sarcopenic patients with HFpEF. On a finishing note, our results also reinforce the need to assess sarcopenia and frailty in HF patients in daily clinical practise and to start planned and personalised nutrition and exercise intervention.

## Conclusion

In resume, being a woman, being older, having HFpEF, using anticoagulants, furosemide, acetylsalicylic acid, and anti-depressants, and not using statins, were factors associated with having concomitant frailty and sarcopenia. A relatively low frequency of coexistence of frailty and sarcopenia means that these conditions still deserve individual attention. Nevertheless, studying this coexistence allowed for isolating the patients who were at higher risk of developing HF complications and, more importantly, pinpointed the relevance of looking more thoroughly at the patients with HFpEF. Pharmacological therapies aimed at this triad of often coexisting conditions of sarcopenia, frailty and HFpEF are still in their emergent stage. For this reason, patients can potentially benefit from interventions that impact their quality of life such as nutritional and mental health interventions and exercise training.

## Data Availability

The data supporting the findings of this study are available from the DeM and AdHeart projects, but restrictions apply to the availability of these data, which were used under license for the current study and are therefore not publicly available. However, the data are available from the corresponding author upon reasonable request and with the permission of the promoting institutions.

## Abbreviations

HF: Heart Failure
NYHA: New York Heart Association
LVEF: Left-Ventricular Ejection Fraction
ESC: European Society of Cardiology
HFrEF: Heart Failure with reduced Ejection Fraction
HFmrEF: Heart Failure with mid-range Ejection Fraction
HFpEF: Heart Failure with preserved Ejection Fraction
EWGSOP2: European Working Group on Sarcopenia in Older People 2 (revised consensus)
SGLT2: Sodium–glucose co-transporter 2
miRNAs: Micro RNAs
